# 4-Methoxy­phenyl 2,3,4,6-tetra-*O*-acetyl-1-thio-α-d-mannopyran­oside

**DOI:** 10.1107/S1600536808019338

**Published:** 2008-07-05

**Authors:** Ludovic Drouin, Andrew R. Cowley, Antony J. Fairbanks, Amber L. Thompson

**Affiliations:** a Department of Chemistry, Chemistry Research Laboratory, University of Oxford, Mansfield Road, Oxford OX1 3TA, England; b Chemical Crystallography Department, Chemistry Research Laboratory, University of Oxford, Mansfield Road, Oxford OX1 3TA, England

## Abstract

The title compound, C_21_H_26_O_10_S, was synthesized in a single step from mannose penta­acetate. The mol­ecular structure confirms the α configuration of the anomeric thioaryl substituent. Spectroscopic and melting-point data obtained for the title compound are in disagreement with those previously reported, indicating the previously reported synthesis [Durette & Shen (1980 ▶). *Carbohydr. Res*. **81**, 261–274] to be erroneous. The crystal structure is stabilized by weak inter­molecular C—H⋯O hydrogen bonds.

## Related literature

For related literature, see: Altomare *et al.* (1994 ▶); Cao *et al.* (1998 ▶); Cosier & Glazer (1986 ▶); Drouin *et al.* (2007 ▶); Durette & Shen (1980 ▶); France *et al.* (2004 ▶); Mootoo *et al.* (1988 ▶); Poh (1982 ▶); Prince (1982 ▶); Roy *et al.* (1992 ▶); Watkin (1994 ▶).
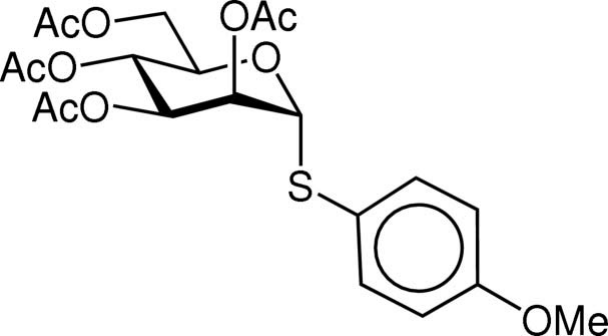


## Experimental

### 

#### Crystal data

C_21_H_26_O_10_S
*M* *_r_* = 470.50
Orthorhombic, 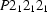

*a* = 8.6218 (2) Å
*b* = 15.2945 (3) Å
*c* = 17.5449 (3) Å
*V* = 2313.58 (8) Å^3^
*Z* = 4
Mo *K*α radiation
μ = 0.19 mm^−1^
*T* = 150 K
0.44 × 0.32 × 0.20 mm

#### Data collection

Nonius KappaCCD diffractometer
Absorption correction: multi-scan *DENZO*/*SCALEPACK* (Otwinowski & Minor, 1997 ▶) *T*_min_ = 0.94, *T*_max_ = 0.96
18167 measured reflections
5253 independent reflections
4562 reflections with *I* > 2.0σ(*I*)
*R*_int_ = 0.033

#### Refinement

*R*[*F* ^2^ > 3σ(*F* ^2^)] = 0.036
*wR*(*F* ^2^) = 0.035
*S* = 1.07
4305 reflections
290 parameters
H-atom parameters constrained
Δρ_max_ = 0.27 e Å^−3^
Δρ_min_ = −0.26 e Å^−3^
Absolute structure: Flack (1983 ▶), 2269 Friedel pairs
Flack parameter: −0.06 (6)

### 

Data collection: *COLLECT* (Nonius, 2001 ▶).; cell refinement: *DENZO*/*SCALEPACK* (Otwinowski & Minor, 1997 ▶); data reduction: *DENZO*/*SCALEPACK* and Hooft *et al.* (2008 ▶); program(s) used to solve structure: *SIR92* (Altomare *et al.*, 1994 ▶); program(s) used to refine structure: *CRYSTALS* (Betteridge *et al.*, 2003 ▶); molecular graphics: *SHELXTL* (Sheldrick, 2008 ▶); software used to prepare material for publication: *CRYSTALS*.

## Supplementary Material

Crystal structure: contains datablocks I, global. DOI: 10.1107/S1600536808019338/lh2646sup1.cif

Structure factors: contains datablocks I. DOI: 10.1107/S1600536808019338/lh2646Isup2.hkl

Additional supplementary materials:  crystallographic information; 3D view; checkCIF report

## Figures and Tables

**Table 1:** Hydrogen-bond geometry (Å, °)

*D*—H⋯*A*	*D*—H	H⋯*A*	*D*⋯*A*	*D*—H⋯*A*
C1—H11⋯O10^i^	0.98	2.40	3.248 (3)	144
C19—H191⋯O2^ii^	0.97	2.54	3.362 (3)	142
C21—H213⋯O6^iii^	0.97	2.43	3.143 (3)	130

Symmetry codes: (i) 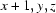; (ii) 
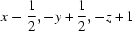
; (iii) 
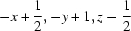
.

**Fig. 1.**
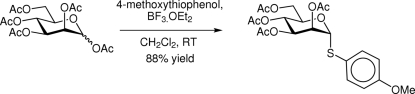
Synthesis of (I).

**Fig. 2.**
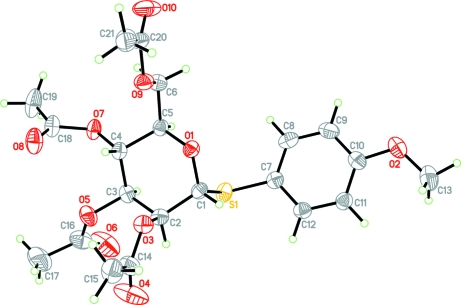
The molecular structure of 4-methoxyphenyl 2,3,4,6-tetra-O-acetyl-1-thio-α-D-mannopyranoside(I) drawn with probability ellipsoids drawn at 50%.

**Fig. 3.**
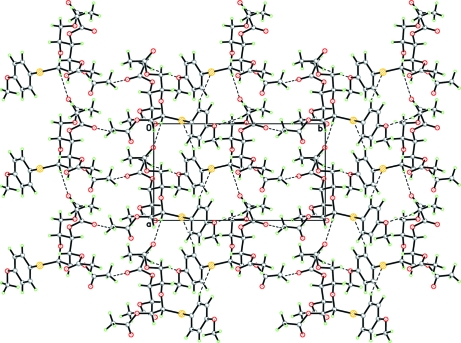
The crystal structure of (I) viewed along the c axis. Intermolecular contacts are shown with a broken line.

## supplementary crystallographic information

### Comment

Thioglycosides are extremely useful and versatile glycoside donors for the synthesis of oligosaccharides, which may be activated by a wide range of electrophiles and also by electrochemical methods (France *et al.*, 2004). The nature of an aromatic substituent of an aryl thioglycoside has a strongly modulating effect on the reactivity of such a thioglycoside; strongly electron donating substituents greatly increase their reactivity towards electrophiles (Roy *et al.*, 1992), and also decrease their oxidation potentials so that they may be electrochemically activated at relatively low externally applied potentials (Drouin *et al.*, 2007). Such 'armed' (Mootoo *et al.*, 1988) thioglycosides may therefore be used as donors for the glycosylation of less reactive 'disarmed' thioglycoside acceptors. The title compound was obtained in a single step from mannose penta-acetate by treatment with 4-methoxythiophenol and boron trifluoride etherate in dichloromethane (Fig. 1). Spectroscopic data obtained for this compound was in disagreement with that previously reported in the only reported synthesis (Durette *et al.*, 1980). Moreover the anomalous optical rotation reported therein had also been highlighted in a subsequent paper (Poh, 1982). Single crystal X-ray analysis was therefore undertaken to confirm the authenticity of our material, and this indeed demonstrated the correctness of our structural assignment (Fig. 2), and in particular the α-anomeric configuration of the thioaryl group. We conclude that the previous report (Durette *et al.*, 1980) in fact probably details the synthesis of the corresponding β-anomer, formed by an S_N_2 substitution reaction on the α-glycosyl bromide, which was incorrectly assigned the α-anomeric configuration by the authors.

The structure has no strong intermolecular interactions, although there are a number of weaker C—H···O interactions that lead to the formation of sheets (Fig. 3 and Table 1)

### Experimental

1,2,3,4,6-Penta-*O*-acetyl-α,β-*D*-mannopyranoside (12.55 g, 32.20 mmol) and 4-methoxythiophenol (5 ml, 40.70 mmol) were suspended in anhydrous dichloromethane (240 ml) under an atmosphere of argon, and the mixture was cooled to 273K. Boron trifluoride diethyl etherate (38.6 ml, 304.60 mmol) was added dropwise, and the reaction mixture was stirred at 295K. After 22 h, t.l.c. (petroleum ether/ethyl acetate, 1:1) indicated the formation of a major product (*R*_f_ 1/2) and the complete consumption of the starting material (*R*_f_ 0.4; 1/2). The reaction was then quenched by the addition of triethylamine and the resulting mixture was partitioned between dichloromethane (240 ml) and water (240 ml). The organic extracts were washed with a saturated aqueous solution of sodium hydrogencarbonate (240 ml), a saturated aqueous solution of sodium chloride (240 ml), and were then dried over MgSO_4_, and concentrated *in vacuo*. The residue was purified by flash column chromatography (petroleum ether/ethyl acetate, 6:4) to give the desired 4-methoxyphenyl 2,3,4,6-tetra-*O*-acetyl-1-thio-α-*D*-mannopyranoside (13.31 g, 88%) which crystallized from cyclohexane as a white crystalline solid, m.p. 335-337K (cyclohexane); a sample suitable for X-ray analysis was then re-crystallized from a solution in pentane/ethyl acetate; [α]_D_^20^ +108 (c, 1.1 in CHCl_3_), [α]_D_^21^ +117 (c, 1.2 in CHCl_3_); 1H (400 MHz, C_6_D_6_) 1.57 (3*H*, s, CH_3_CO), 1.67 (3*H*, s, CH_3_CO), 1.69 (3*H*, s, CH_3_CO), 1.70 (3*H*, s, CH_3_CO), 3.28 (3*H*, s, OCH_3_), 4.17 (1*H*, dd, J_5,6_ 2.5 Hz, J_6,6'_ 12.5 Hz, H-6), 4.42 (1*H*, dd, J_5,6'_ 5.5 Hz, J_6,6'_ 12.5 Hz, H-6'), 4.65 (1*H*, ddd, J_4,5_ 8.0 Hz, J_5,6_ 2.5 Hz, J_5,6'_ 5.5 Hz, H-5), 5.42 (1*H*, brs, CH), 5.70–5.80 (2 *x* 1H, m, 2 *x* CH), 5.87 (1*H*, brs, CH), 6.60 (2 *x* 1H, dd, J 9.0 Hz, J 0.5 Hz, 2ArH), 7.34 (2 *x* 1H, dd, J 9.0 Hz, J 0.5 Hz, 2ArH); δ_H_ (400 MHz, CDCl_3_) 2.02 (3*H*, s, CH_3_CO), 2.08 (2 *x* 3H, s, 2 *x* CH_3_CO), 2.15 (3*H*, s, CH_3_CO), 3.80 (3*H*, s, OCH_3_), 4.12 (1*H*, dd, J_6,6'_ 12.0 Hz, J_5,6_ 2.0 Hz, H-6), 4.31 (1*H*, dd, J_6,6'_ 4.0 Hz, J_5,6'_ 6.0 Hz, H-6'), 4.58 (1*H*, ddd, J_5,6_ 2.0 Hz, J_5,6'_ 6.0 Hz, J_4,5_ 10.0 Hz, H-5), 5.31–5.34 (3 *x* 1H, m, H-1, 2 *x* CH), 5.50 (1*H*, brs, CH), 6.86 (2 *x* 1H, dd, J 9.0 Hz, J 1.5 Hz, ArH), 7.43 (2 *x* 1H, dd, J 9.0 Hz, J 1.5 Hz, ArH); δ_C_ (50 MHz, CDCl_3_) 20.8 (CH_3_CO), 20.9 (2 *x* CH_3_CO), 21.0 (CH_3_CO), 55.5 (CH_3_O), 62.7 (C-6), 66.6 (CH), 69.5 (2 *x* CH), 70.89 (CH), 86.7 (C-1), 114.9 (2 *x* ArCH), 122.7 (ArC), 135.2 (2 *x* ArCH), 160.3 (ArC), 169.9 (C?O), 169.9 (C?O), 170.1 (C?O), 170.7 (C?O); m/*z* (ESI) 529.37 ([*M*+NH_4_+CH_3_CN]^+^, 100%); (HMRS (ESI) Calcd. For C_21_H_26_NaO_10_S (*M*+NH_4_^+^) 493.1139. Found 493.1127).

### Refinement

A polycrystalline aggregate was divided to give a fragment having dimensions approximately  0.2 *x* 0.32 *x* 0.44 mm, which was mounted on a glass fibre using perfluoropolyether oil. The sample was cooled rapidly to 150 K in a stream of cold N_2_ using an Oxford Cryosystems Cryostream unit (Cosier and Glazer, 1986). Diffraction data were measured using an Bruker–Nonius KappaCCD diffractometer (graphite-monochromated Mo *K*α radiation, λ = 0.71073 Å). Intensity data were processed using the *DENZO-SMN* package (Otwinowski and Minor, 1997).

Examination of the systematic absences of the intensity data showed the space group to be P2_1_2_1_2_1_ and the structure was solved using the direct-methods program *SIR92* (Altomare *et al.*, 1994), which located all ordered non-hydrogen atoms. Subsequent full-matrix least-squares refinement was carried out using the *CRYSTALS* program suite (Betteridge *et al.*, 2003). Coordinates and anisotropic thermal parameters of all non-hydrogen atoms were refined. The relatively large thermal parameters of some of the acetate carbon and carbonyl oxygen atoms (Figure 1) suggest that there may be unresolved disorder of these groups. Attempts to model this did not lead to any improvement in the agreement with the X-ray data and were abandoned.

Refinement of the Flack *x* parameter (Flack, 1983) gave a value of -0.063 (63) and examination of the Bijvoet Pairs gave the Hooft *y* parameter as -0.016 (29) (G=1.031 (59)) and giving the probability that the absolute configuration is correct as 1.000, using either a two or three-hypothesis model (Hooft *et al.*, 2008).

The hydrogen atoms were all visible in the difference map, but were repositioned geometrically. Initially they were refined with soft restraints on the bond lengths and angles to regularize their geometry (C—H in the range 0.93–0.98), and *U*_iso_(H) (in the range 1.2–1.5 times *U*_eq_ of the parent atom), after which the positions were refined with riding constraints.

A 3-term Chebychev polynomial weighting scheme was applied w = [1-(||F_o_|-F_c_||/6σ(F_o_))^2^]^2^ / [0.350T_0_(x)+0.0808T_1_(x) + 0.0749]*T_n-1_(x)] (Watkin, 1994, Prince, 1982) and the refinement was carried out using a 3 σ  cutoff giving a total of 4305 reflections.

### Crystal data

**Table:**

C_21_H_26_O_10_S_1_	*D*_x_ = 1.351 Mg m^−^^3^
*M**_r_* = 470.50	Melting point: not measured K
Orthorhombic, *P*2_1_2_1_2_1_	Mo *K*α radiation λ = 0.71073 Å
Hall symbol: P 2ac 2ab	Cell parameters from 18167 reflections
*a* = 8.6218 (2) Å	θ = 5–28º
*b* = 15.2945 (3) Å	µ = 0.19 mm^−^^1^
*c* = 17.5449 (3) Å	*T* = 150 K
*V* = 2313.58 (8) Å^3^	Fragment, colourless
*Z* = 4	0.44 × 0.32 × 0.20 mm
*F*_000_ = 992	

### Data collection

**Table:**

Area diffractometer	4562 reflections with *I* > 2.0σ(*I*)
Monochromator: graphite	*R*_int_ = 0.033
*T* = 150 K	θ_max_ = 27.5º
ω scans	θ_min_ = 5.2º
Absorption correction: multi-scanDENZO/SCALEPACK (Otwinowski & Minor, 1997)	*h* = −11→11
*T*_min_ = 0.94, *T*_max_ = 0.96	*k* = −19→19
18167 measured reflections	*l* = −22→22
5253 independent reflections	

### Refinement

**Table:**

Refinement on *F*	H-atom parameters constrained
Least-squares matrix: full	Method, part 1, Chebychev polynomial, (Watkin, 1994, *P*rince, 1982) [*w*eight] = 1.0/[A_0_*T_0_(x) + A_1_*T_1_(x) ··· + A_n-1_]*T_n-1_(x)] where A_i_ are the Chebychev coefficients listed belo*w* and x = *F* /*F*max Method = Robust Weighting (*P*rince, 1982) W = [*w*eight] * [1-(delta*F*/6*sigma*F*)^2^]^2^ A_i_ are: 0.350 0.808E-01 0.749E-01
*R*[*F*^2^ > 2σ(*F*^2^)] = 0.036	(Δ/σ)_max_ = 0.001
*wR*(*F*^2^) = 0.035	Δρ_max_ = 0.27 e Å^−^^3^
*S* = 1.07	Δρ_min_ = −0.26 e Å^−^^3^
4305 reflections	Extinction correction: None
290 parameters	Absolute structure: Flack (1983), 2269 Friedel pairs
Primary atom site location: structure-invariant direct methods	Flack parameter: −0.06 (6)
Hydrogen site location: inferred from neighbouring sites	

### Special details

**Table:**

Refinement. The hydrogen atoms were all located in a difference map, but those attached to carbon atoms were repositioned geometrically. The H atoms were initially refined with soft restraints on the bond lengths and angles to regularize their geometry (C—H in the range 0.93–0.98, N—H in the range 0.86–0.89, N—H to 0.86, O—H = 0.82 Å) and *U*_iso_(H) (in the range 1.2–1.5 times *U*_eq_ of the parent atom), after which the positions were refined with riding constraints.

### Fractional atomic coordinates and isotropic or equivalent isotropic displacement parameters (Å^2^)

**Table:**

	*x*	*y*	*z*	*U*_iso_*/*U*_eq_	
C1	0.42086 (19)	0.44662 (10)	0.48435 (10)	0.0278	
C2	0.4623 (2)	0.51157 (10)	0.54678 (9)	0.0290	
C3	0.3411 (2)	0.51009 (11)	0.60972 (9)	0.0293	
C4	0.1802 (2)	0.52268 (11)	0.57752 (9)	0.0272	
C5	0.15111 (19)	0.45186 (11)	0.51798 (10)	0.0276	
C6	−0.0057 (2)	0.45811 (11)	0.48018 (11)	0.0333	
O1	0.26620 (14)	0.45701 (8)	0.45874 (7)	0.0279	
S1	0.46552 (6)	0.33607 (3)	0.51937 (3)	0.0339	
C7	0.4854 (2)	0.27972 (11)	0.43134 (10)	0.0306	
C8	0.3607 (2)	0.23433 (13)	0.40015 (12)	0.0390	
C9	0.3778 (2)	0.19047 (13)	0.33162 (13)	0.0415	
C10	0.5189 (2)	0.19112 (11)	0.29336 (10)	0.0336	
C11	0.6441 (2)	0.23463 (12)	0.32488 (10)	0.0329	
C12	0.6267 (2)	0.27925 (12)	0.39370 (11)	0.0317	
O2	0.52304 (19)	0.14679 (9)	0.22609 (8)	0.0434	
C13	0.6652 (3)	0.14715 (16)	0.18392 (12)	0.0527	
O3	0.46128 (15)	0.59743 (7)	0.51222 (7)	0.0317	
C14	0.5794 (2)	0.65171 (13)	0.52960 (12)	0.0401	
O4	0.6831 (2)	0.63201 (11)	0.57186 (12)	0.0741	
C15	0.5639 (3)	0.73699 (13)	0.48905 (13)	0.0471	
O5	0.36891 (17)	0.58054 (9)	0.66273 (7)	0.0383	
C16	0.4478 (3)	0.56208 (15)	0.72635 (11)	0.0448	
O6	0.5010 (3)	0.49096 (12)	0.73892 (11)	0.0767	
C17	0.4616 (4)	0.6411 (2)	0.77608 (14)	0.0696	
O7	0.06770 (15)	0.50827 (8)	0.63709 (7)	0.0334	
C18	0.0046 (2)	0.58014 (12)	0.67193 (10)	0.0374	
O8	0.04098 (19)	0.65349 (8)	0.65627 (8)	0.0466	
C19	−0.1121 (3)	0.55302 (15)	0.72963 (15)	0.0595	
O9	−0.01388 (15)	0.54017 (8)	0.43982 (7)	0.0341	
C20	−0.1484 (2)	0.55419 (14)	0.40247 (11)	0.0378	
O10	−0.25415 (18)	0.50261 (13)	0.40449 (10)	0.0578	
C21	−0.1459 (3)	0.63787 (15)	0.35898 (12)	0.0476	
H11	0.4895	0.4569	0.4405	0.0329*	
H21	0.5668	0.4991	0.5672	0.0352*	
H31	0.3454	0.4533	0.6367	0.0357*	
H41	0.1694	0.5812	0.5567	0.0321*	
H51	0.1563	0.3935	0.5428	0.0332*	
H61	−0.0883	0.4553	0.5182	0.0412*	
H62	−0.0189	0.4084	0.4439	0.0426*	
H81	0.2625	0.2331	0.4264	0.0476*	
H91	0.2901	0.1590	0.3105	0.0504*	
H111	0.7447	0.2344	0.2983	0.0390*	
H121	0.7146	0.3117	0.4155	0.0391*	
H131	0.6468	0.1130	0.1371	0.0801*	
H132	0.7494	0.1231	0.2157	0.0787*	
H133	0.6895	0.2084	0.1693	0.0802*	
H152	0.6314	0.7806	0.5124	0.0710*	
H151	0.4574	0.7570	0.4924	0.0705*	
H153	0.5884	0.7284	0.4351	0.0710*	
H172	0.5416	0.6301	0.8131	0.1035*	
H171	0.3633	0.6515	0.7991	0.1058*	
H173	0.4931	0.6909	0.7446	0.1044*	
H192	−0.1471	0.6041	0.7564	0.0883*	
H191	−0.0655	0.5113	0.7647	0.0876*	
H193	−0.2004	0.5241	0.7047	0.0882*	
H212	−0.2487	0.6498	0.3385	0.0716*	
H211	−0.1161	0.6835	0.3930	0.0723*	
H213	−0.0724	0.6340	0.3170	0.0725*	

### Atomic displacement parameters (Å^2^)

**Table:**

	*U*^11^	*U*^22^	*U*^33^	*U*^12^	*U*^13^	*U*^23^
C1	0.0262 (8)	0.0281 (8)	0.0291 (8)	0.0025 (6)	0.0019 (7)	0.0036 (7)
C2	0.0285 (8)	0.0272 (8)	0.0312 (8)	0.0019 (7)	−0.0038 (8)	0.0055 (6)
C3	0.0376 (9)	0.0248 (8)	0.0256 (8)	0.0018 (7)	−0.0030 (7)	0.0019 (6)
C4	0.0311 (9)	0.0255 (8)	0.0250 (8)	0.0008 (7)	0.0039 (7)	0.0025 (6)
C5	0.0281 (8)	0.0269 (8)	0.0277 (8)	−0.0001 (6)	0.0036 (7)	0.0005 (7)
C6	0.0315 (9)	0.0336 (8)	0.0349 (8)	−0.0036 (7)	−0.0019 (8)	0.0011 (8)
O1	0.0282 (6)	0.0299 (6)	0.0258 (6)	0.0020 (5)	0.0010 (5)	0.0012 (5)
S1	0.0391 (2)	0.03005 (19)	0.0327 (2)	0.00833 (19)	0.0037 (2)	0.00485 (18)
C7	0.0322 (9)	0.0244 (7)	0.0352 (9)	0.0038 (7)	−0.0009 (8)	0.0044 (6)
C8	0.0267 (9)	0.0412 (10)	0.0492 (11)	0.0006 (8)	0.0011 (8)	0.0039 (9)
C9	0.0348 (10)	0.0409 (10)	0.0488 (11)	−0.0048 (8)	−0.0094 (9)	−0.0022 (9)
C10	0.0397 (10)	0.0271 (8)	0.0342 (9)	0.0012 (8)	−0.0072 (8)	0.0003 (7)
C11	0.0325 (9)	0.0310 (9)	0.0354 (9)	−0.0011 (7)	0.0044 (8)	0.0002 (7)
C12	0.0310 (9)	0.0275 (8)	0.0367 (9)	−0.0022 (7)	−0.0006 (8)	0.0023 (7)
O2	0.0544 (9)	0.0377 (7)	0.0380 (7)	0.0002 (7)	−0.0072 (7)	−0.0076 (6)
C13	0.0666 (15)	0.0549 (13)	0.0365 (11)	0.0092 (12)	−0.0010 (11)	−0.0083 (10)
O3	0.0334 (6)	0.0279 (6)	0.0338 (6)	−0.0016 (5)	−0.0016 (6)	0.0066 (5)
C14	0.0412 (10)	0.0334 (9)	0.0457 (11)	−0.0063 (8)	−0.0018 (9)	−0.0007 (8)
O4	0.0709 (12)	0.0497 (10)	0.1018 (15)	−0.0241 (9)	−0.0468 (12)	0.0199 (10)
C15	0.0560 (13)	0.0304 (9)	0.0549 (12)	−0.0044 (9)	0.0132 (11)	0.0037 (9)
O5	0.0513 (8)	0.0370 (7)	0.0266 (6)	0.0028 (6)	−0.0104 (6)	−0.0042 (5)
C16	0.0469 (12)	0.0581 (13)	0.0293 (9)	−0.0087 (10)	−0.0104 (9)	0.0065 (9)
O6	0.1008 (16)	0.0624 (11)	0.0669 (11)	−0.0045 (11)	−0.0511 (12)	0.0164 (9)
C17	0.0801 (19)	0.0841 (18)	0.0445 (12)	−0.0086 (16)	−0.0198 (14)	−0.0186 (12)
O7	0.0412 (7)	0.0274 (6)	0.0316 (6)	0.0034 (5)	0.0126 (5)	−0.0009 (5)
C18	0.0477 (11)	0.0303 (9)	0.0341 (9)	0.0071 (8)	0.0074 (9)	−0.0021 (7)
O8	0.0655 (10)	0.0284 (6)	0.0460 (8)	0.0065 (7)	0.0131 (8)	0.0003 (6)
C19	0.0782 (17)	0.0413 (12)	0.0591 (14)	0.0126 (12)	0.0369 (14)	0.0011 (10)
O9	0.0268 (6)	0.0367 (6)	0.0387 (7)	0.0021 (5)	−0.0064 (5)	0.0006 (5)
C20	0.0249 (9)	0.0564 (12)	0.0321 (9)	0.0068 (9)	−0.0045 (8)	−0.0100 (8)
O10	0.0281 (7)	0.0905 (13)	0.0547 (10)	−0.0112 (7)	−0.0078 (7)	0.0022 (9)
C21	0.0476 (12)	0.0529 (13)	0.0423 (11)	0.0190 (10)	−0.0134 (10)	−0.0079 (9)

### Geometric parameters (Å, °)

**Table:**

C1—C2	1.521 (2)	C12—H121	0.984
C1—O1	1.416 (2)	O2—C13	1.432 (3)
C1—S1	1.8398 (16)	C13—H131	0.985
C1—H11	0.984	C13—H132	0.987
C2—C3	1.520 (2)	C13—H133	0.994
C2—O3	1.4464 (18)	O3—C14	1.349 (2)
C2—H21	0.988	C14—O4	1.200 (3)
C3—C4	1.511 (2)	C14—C15	1.492 (3)
C3—O5	1.443 (2)	C15—H152	0.975
C3—H31	0.990	C15—H151	0.970
C4—C5	1.525 (2)	C15—H153	0.978
C4—O7	1.443 (2)	O5—C16	1.337 (2)
C4—H41	0.972	C16—O6	1.201 (3)
C5—C6	1.509 (2)	C16—C17	1.496 (3)
C5—O1	1.439 (2)	C17—H172	0.962
C5—H51	0.995	C17—H171	0.952
C6—O9	1.443 (2)	C17—H173	0.980
C6—H61	0.977	O7—C18	1.370 (2)
C6—H62	0.998	C18—O8	1.197 (2)
S1—C7	1.7770 (18)	C18—C19	1.486 (3)
C7—C8	1.392 (3)	C19—H192	0.961
C7—C12	1.385 (3)	C19—H191	0.973
C8—C9	1.385 (3)	C19—H193	0.983
C8—H81	0.965	O9—C20	1.350 (2)
C9—C10	1.390 (3)	C20—O10	1.206 (3)
C9—H91	0.969	C20—C21	1.490 (3)
C10—C11	1.384 (3)	C21—H212	0.973
C10—O2	1.362 (2)	C21—H211	0.953
C11—C12	1.395 (3)	C21—H213	0.974
C11—H111	0.984		
			
C2—C1—O1	112.11 (13)	C12—C11—H111	120.4
C2—C1—S1	108.09 (12)	C11—C12—C7	120.66 (17)
O1—C1—S1	113.96 (11)	C11—C12—H121	120.0
C2—C1—H11	108.5	C7—C12—H121	119.3
O1—C1—H11	107.4	C10—O2—C13	117.93 (17)
S1—C1—H11	106.4	O2—C13—H131	106.9
C1—C2—C3	110.61 (14)	O2—C13—H132	109.7
C1—C2—O3	106.83 (13)	H131—C13—H132	113.0
C3—C2—O3	108.29 (13)	O2—C13—H133	108.5
C1—C2—H21	110.4	H131—C13—H133	108.5
C3—C2—H21	111.1	H132—C13—H133	110.1
O3—C2—H21	109.4	C2—O3—C14	117.36 (14)
C2—C3—C4	110.96 (14)	O3—C14—O4	123.21 (18)
C2—C3—O5	110.06 (14)	O3—C14—C15	111.29 (17)
C4—C3—O5	107.35 (14)	O4—C14—C15	125.49 (19)
C2—C3—H31	109.6	C14—C15—H152	110.1
C4—C3—H31	108.9	C14—C15—H151	109.4
O5—C3—H31	109.9	H152—C15—H151	108.9
C3—C4—C5	108.45 (13)	C14—C15—H153	109.0
C3—C4—O7	109.09 (13)	H152—C15—H153	111.7
C5—C4—O7	106.10 (13)	H151—C15—H153	107.8
C3—C4—H41	110.2	C3—O5—C16	117.69 (15)
C5—C4—H41	112.4	O5—C16—O6	122.6 (2)
O7—C4—H41	110.4	O5—C16—C17	110.9 (2)
C4—C5—C6	113.78 (14)	O6—C16—C17	126.5 (2)
C4—C5—O1	110.03 (13)	C16—C17—H172	108.0
C6—C5—O1	107.28 (14)	C16—C17—H171	108.1
C4—C5—H51	109.3	H172—C17—H171	112.4
C6—C5—H51	106.9	C16—C17—H173	108.7
O1—C5—H51	109.5	H172—C17—H173	108.6
C5—C6—O9	108.34 (13)	H171—C17—H173	110.9
C5—C6—H61	110.6	C4—O7—C18	117.88 (13)
O9—C6—H61	109.7	O7—C18—O8	123.04 (17)
C5—C6—H62	109.5	O7—C18—C19	110.42 (16)
O9—C6—H62	110.1	O8—C18—C19	126.54 (18)
H61—C6—H62	108.6	C18—C19—H192	108.6
C5—O1—C1	114.46 (13)	C18—C19—H191	109.5
C1—S1—C7	100.12 (8)	H192—C19—H191	110.7
S1—C7—C8	120.59 (14)	C18—C19—H193	110.3
S1—C7—C12	120.12 (14)	H192—C19—H193	109.9
C8—C7—C12	119.28 (17)	H191—C19—H193	107.8
C7—C8—C9	120.06 (18)	C6—O9—C20	114.75 (14)
C7—C8—H81	120.0	O9—C20—O10	122.1 (2)
C9—C8—H81	119.9	O9—C20—C21	111.87 (17)
C8—C9—C10	120.60 (18)	O10—C20—C21	126.01 (19)
C8—C9—H91	119.3	C20—C21—H212	109.7
C10—C9—H91	120.1	C20—C21—H211	108.2
C9—C10—C11	119.57 (17)	H212—C21—H211	109.9
C9—C10—O2	115.99 (17)	C20—C21—H213	110.2
C11—C10—O2	124.43 (18)	H212—C21—H213	108.9
C10—C11—C12	119.80 (18)	H211—C21—H213	110.0
C10—C11—H111	119.7		

### Hydrogen-bond geometry (Å, °)

**Table:**

*D*—H···*A*	*D*—H	H···*A*	*D*···*A*	*D*—H···*A*
C1—H11···O10^i^	0.98	2.40	3.248 (3)	144
C19—H191···O2^ii^	0.97	2.54	3.362 (3)	142
C21—H213···O6^iii^	0.97	2.43	3.143 (3)	130

Symmetry codes: (i) *x*+1, *y*, *z*; (ii) *x*−1/2, −*y*+1/2, −*z*+1; (iii) −*x*+1/2, −*y*+1, *z*−1/2.
